# Elevated Oxidative Stress and Inflammation in Hypothalamic Paraventricular Nucleus Are Associated With Sympathetic Excitation and Hypertension in Rats Exposed to Chronic Intermittent Hypoxia

**DOI:** 10.3389/fphys.2018.00840

**Published:** 2018-07-05

**Authors:** Tiejun Li, Yanli Chen, Chaojun Gua, Baogang Wu

**Affiliations:** ^1^Department of Cardiology, Shengjing Hospital of China Medical University, Shenyang, China; ^2^Department of Cardiology, The First Hospital of China Medical University, Shenyang, China

**Keywords:** obstructive sleep apnea, sympathetic excitation, hypertension, oxidative stress, neuroinflammation

## Abstract

Obstructive sleep apnea (OSA), characterized by recurrent collapse of the upper airway during sleep leading to chronic intermittent hypoxia (CIH), is an independent risk factor for hypertension. Sympathetic excitation has been shown to play a major role in the pathogenesis of OSA-associated hypertension. Accumulating evidence indicates that oxidative stress and inflammation in the hypothalamic paraventricular nucleus (PVN), a critical cardiovascular and autonomic center, mediate sympathetic excitation in many cardiovascular diseases. Here we tested the hypothesis that CIH elevates oxidative stress and inflammation in the PVN, which might be associated with sympathetic excitation and increased blood pressure in a rat model of CIH that mimics the oxygen profile in patients with OSA. Sprague-Dawley rats were pretreated with intracerebroventricular (ICV) infusion of vehicle or superoxide scavenger tempol, and then exposed to control or CIH for 7 days. Compared with control+vehicle rats, CIH+vehicle rats exhibited increased blood pressure, and increased sympathetic drive as indicated by the blood pressure response to ganglionic blockade and plasma norepinephrine levels. Pretreatment with ICV tempol prevented CIH-induced increases in blood pressure and sympathetic drive. Molecular studies revealed that expression of NAD(P)H oxidase subunits, production of reactive oxygen species, expression of proinflammatory cytokines and neuronal excitation in the PVN were elevated in CIH+vehicle rats, compared with control+vehicle rats, but were normalized or reduced in CIH rat pretreated with ICV tempol. Notably, CIH+vehicle rats also had increased systemic oxidative stress and inflammation, which were not altered by ICV tempol. The results suggest that CIH induces elevated oxidative stress and inflammation in the PVN, which lead to PVN neuronal excitation and are associated with sympathetic excitation and increased blood pressure. Central oxidative stress and inflammation may be novel targets for the prevention and treatment of hypertension in patients with OSA.

## Introduction

Obstructive sleep apnea (OSA) is a medical condition characterized by repetitive nocturnal upper airway obstructions during sleep leading to chronic intermittent hypoxia (CIH) and sleep fragmentation (Ravensbergen and Sahota, 2009; Hinkle et al., 2018). This condition has strong epidemiologic associations with cardiovascular disease and has been shown to be an independent risk factor for hypertension (Ravensbergen and Sahota, 2009; Hinkle et al., 2018). It is estimated that more than 50% of patients with sleep apnea are clinically hypertensive (Millman et al., 1991; Parati et al., 2007; Fusetti et al., 2012). Consistent with clinical observations, experimental studies in animals have revealed a similar increase in blood pressure after as few as 3–7 days of CIH, which mimics the episodes of hypoxemia that occur in individuals with OSA (Hinojosa-Laborde and Mifflin, 2005; Sharpe et al., 2013). Considerable evidence has suggested that increased sympathetic nervous system activity plays a key role in both initiating and maintaining chronic increase of blood pressure. For example, patients with OSA exhibit sympathetic excitation that persists even beyond the sleep period (Carlson et al., 1993). Animal studies also show that CIH increases sympathetic nerve activity and results in sustained increase of blood pressure (Fletcher, 2001; Sharpe et al., 2013). Interventions to inhibit sympathetic nerve activity prevent CIH-induced increase in blood pressure (Sunderram and Androulakis, 2012; Carmichael and Wainford, 2015).

The paraventricular nucleus (PVN) of the hypothalamus is an important forebrain center that integrates and responds to a variety of neural and humoral signals regulating sympathetic drive and extracellular fluid volume status (Badoer, 2010; Zheng and Patel, 2017). Activation of the PVN has been shown to increase sympathetic nerve activity and blood pressure in rats exposed to CIH (Knight et al., 2011; Sharpe et al., 2013). Accumulating evidence indicates that oxidative stress and inflammation in the PVN are major contributors to the augmented sympathetic nerve activity in various forms of hypertension and other cardiovascular diseases (Dange et al., 2015; Khor and Cai, 2017; Haspula and Clark, 2018; Jiang et al., 2018; Zhang et al., 2018). A recent study has demonstrated that CIH causes elevated oxidative stress and inflammation in the brain regions associated with neurodegeneration (Snyder et al., 2017). To date, however, no studies have examined the role of oxidative stress and inflammation in the PVN in regulation of sympathetic nerve activity and blood pressure in either patients with OSA or in animal models of CIH. Here we tested the hypothesis that CIH induces elevation of oxidative stress and inflammation in the PVN, which might be associated with increased sympathetic nerve activity and blood pressure. For this purpose, we used a rat model of CIH that closely mimics the oxygen profile in patients with OSA.

## Materials and Methods

### Animals

Male Sprague-Dawley rats at 10 weeks of age (body weight: 300–325 g) were purchased from Vital River (A Charles River Company, Beijing, China). All animals were individually housed at 22 ± 1°C with a controlled 12:12 h light/dark cycle and free access to food and water. All experiments were approved by the Institutional Animal Care and Use Committee of China Medical University and were performed according to the “Guiding Principles for Research Involving Animals and Human Beings.”

### Protocols

Thirty-two rats were anesthetized and a telemetry transmitter was implanted in the femoral artery for continuous monitoring of mean blood pressure (MBP) and heart rate (HR). Five days after recovery from surgery, these rats and additional 40 rats without implantation of telemetry transmitters underwent intracerebroventricular (ICV) cannula and osmotic pump implantation for chronic infusion of tempol (TEM) or vehicle (VEH). Seven days later, all animals were exposed to CIH or control (CON) for 7 days. Since CIH exposure for only 3 days could significantly increase blood pressure in rats (Sharpe et al., 2013), we therefore started to treat animals with tempol at 7 days prior to CIH to ensure that CIH-induced oxidative stress could be completely prevented before blood pressure change. Telemetry signals were recorded 5 days before (baseline) and throughout the 7-day CIH/control protocol. MBP and HR obtained from telemetry signals were determined as the mean of 24-h averages. One day prior to sacrifice, animal with implantation of telemetry transmitters received the ganglionic blocker hexamethonium bromide (30 mg/kg ip) (Xue et al., 2011) to evaluate the sympathetic contribution to MBP. These rats received the CIH exposure until the end of the study protocol. Seven days after CIH, animals with implantation of telemetry transmitters and receiving hexamethonium bromide injection were sacrificed to collect brains and blood for molecular and biochemical analyses. Other animals without implantation of telemetry transmitters and hexamethonium bromide injection were perfused transcardially with 4% paraformaldehyde for immunohistochemical study or directly collected brain for detection of reactive oxygen species (ROS). The interval between intermittent hypoxia and animal sacrifice was 1–3 h.

### Implantation of Telemetry Transmitters

Telemetry transmitter implantation was performed as previously described (Xue et al., 2011). Briefly, rats were anesthetized with a ketamine-xylazine mixture (100 and 10 mg/kg). Under sterile conditions, the right femoral artery was isolated and a telemetry transmitter (TA11PA-C40, Data Science International, MN, United States) was implanted in the artery. The body of the transmitter was placed subcutaneously in the right flank.

### ICV Cannula and Osmotic Pump Implantation

Animals were anesthetized with a ketamine-xylazine mixture. Under sterile conditions, a cannula was implanted into a lateral cerebral ventricle (the coordinates were 0.9 mm caudal, 1.5 mm lateral to bregma, and 4.5 mm below the skull surface), as previously described (Xue et al., 2011). At the same time, an osmotic mini-pump (model 2002; Alzet) for central infusion of tempol (TEM, a superoxide scavenger, 5 μg/min, 6 μg/μl in 200 μl) or vehicle was implanted under the skin on the back of the animal and connected to the cannula with silastic tubing. The tempol dose for ICV infusion was based on a previous study showing optimal *in vivo* prevention of hypothalamic oxidative stress in rats (Toklu et al., 2017). At the end of the experiment, the osmotic mini-pumps were removed to check residual volume to ensure that the drug was entirely infused. Appropriate cannula placement was verified by sectioning the brain to check needle tracks.

### Exposure to CIH

Animals receiving ICV tempol or vehicle infusion were individually placed in the custom-manufactured chambers equipped with the intermittent hypoxia apparatus. Animals assigned to CIH group were exposed to CIH for 8 h/day from 08:00 to16:00 h for 7 consecutive days, as previously described (Sharpe et al., 2013). CIH was carried out during the 12 h of light cycle to coincide with the animal sleep cycle. During CIH exposure, the oxygen concentration inside the chamber was repetitively cycled between 21 and 10% with a period of 6 min (10 cycles/h). Rats assigned to control group were only exposed to room air (21% O^2^) throughout the 7-day protocol. One day prior to sacrifice, the ganglionic blocker hexamethonium bromide (30 mg/kg, i.p.) was given to those animals with implantation of telemetry transmitters to determine the sympathetic contribution to MBP, as previously described (Xue et al., 2011).

### Immunohistochemistry

Animals were deeply anesthetized with 5% isoflurane and then transcardiacally perfused with saline followed by 4% phosphate-buffered paraformaldehyde. Brains were removed and postfixed for 24 h in 4% paraformaldehyde and then equilibrated in 30% sucrose solution at 4°C. Coronal sections were cut at 18 μm using a cryostat and then mounted on glass microscope slides (Fisherbrand^®^ Superfrost Plus, Fisher Scientific, Pittsburgh, PA, United States). C-Fos activity, a marker of neuronal activation, was detected in the PVN using a rabbit anti-c-Fos antibody (c-fos K-25; Santa Cruz Biotechnology, Santa Cruz, CA, United States) as previously described (Bai et al., 2017). In each animal, c-Fos positive neurons within a window (4 × 10^4^ μm^2^) superimposed over the posterior magnocellular, ventrolateral parvocellular, medial parvocellular, and dorsal parvocellular subregions of PVN were counted manually in two representative coronal sections approximately −1.8 mm from bregma and were averaged for data analysis.

### Western Blot Analysis

**FIGURE 1:**
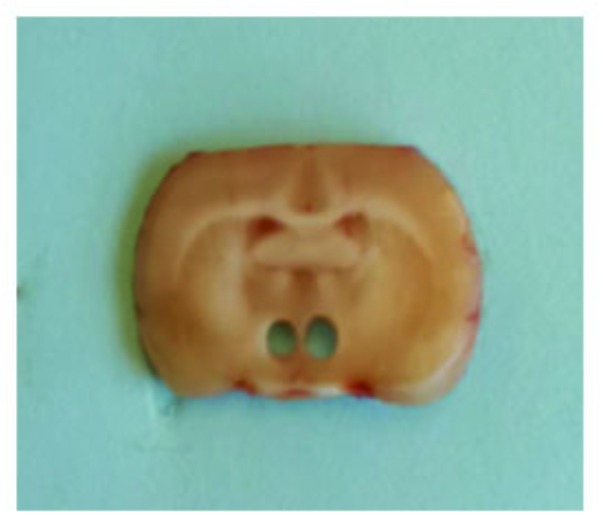
Slice of forebrain illustrating the location of the micropunches obtained from the paraventricular nucleus (PVN) and used for molecular analysis.

The PVN tissues were obtained using a micropunch technique as previously described (Durgam and Mifflin, 1998). Briefly, the brains were quickly removed, frozen in liquid nitrogen and stored at −80°C until ready for use. Using a rat brain slicer (Braintree Scientific Inc, Braintree, MA, United States), 1-mm thick sections were obtained from the PVN of the hypothalamus. Using the third ventricle as an anatomical landmark, bilateral PVN micropunches were taken from each brain (**Figure 1**) and placed in tissue lysis buffer with protease inhibitor (Sigma-Aldrich, St. Louis, MO, United States). PVN tissues were homogenized and the protein concentrations were measured with the Bradford method. An equal amount of protein for each sample was loaded in 12% SDS-polyacrylamide gels. After electrophoresis, the proteins were transferred to polyvinylidene difluoride membranes and blocked with 2% skimmed milk in Tris-buffered saline containing 0.1% Tween 20. The membranes were incubated with primary antibodies to NAD(P)H oxidase subunits p47^phox^ and gp91^phox^ (Santa Cruz Biotechnology, Santa Cruz, CA, United States), pro-inflammatory cytokines tumor necrosis factor (TNF)-α, interleukin (IL)-1β and IL-6 (Cell Signaling Technology, Beverly, MA, United States), and β-actin (Santa Cruz Biotechnology, Santa Cruz, CA, United States) overnight at 4°C. After incubation with HRP-conjugated secondary antibody (Santa Cruz Biotechnology, Santa Cruz, CA, United States) at room temperature for 1 h, immunoreactivity was visualized by the ECL Plus detection system (GE Healthcare, Piscataway, NJ, United States) and then quantified by ImageJ software (National Institutes of Health, Bethesda, MD, United States). All data were corrected by β-actin.

### Detection of ROS

Dihydroethidium (DHE), an oxidative fluorescent dye, was used to evaluate ROS production in the PVN as described previously (Li T. et al., 2017). Briefly, the brain was immediately frozen at −80°C for 1 h and then cut into 10 μm-thick coronal sections with a cryostat. The brain sections were mounted on microscope slides and incubated with DHE (2 μmol/L, Molecular Probes, Grand Island, NY, United States) for 30 min at 37°C in a light-protected humidified chamber. Images were acquired using a Zeiss fluorescence microscope and fluorescence in posterior magnocellular, dorsal parvocellular, medial parvocellular, and ventrolateral parvocellular of the PVN were analyzed by image J software (NIH, Bethesda, MD, United States).

### Biochemical Measurements

At the termination of the experiment, rats were decapitated and the trunk blood was collected into prechilled 10-mL tubes containing EDTA. Plasma was separated by centrifugation for 15 min at 3000 rpm. The level of norepinephrine (NE) in plasma was measured with an ELISA kit (Rocky Mountain Diagnostics, Inc, Colorado Springs, United States). The level of the advanced oxidation protein products (AOPP) in plasma was determined with an OxiSelect^TM^ AOPP Assay Kit (Cell Biolabs, Inc. San Diego, CA, United States). The levels of IL-1β and IL-6 in plasma were analyzed with ELISA kits (Thermo Fisher Scientific, Grand Island, NY, United States).

### Statistical Analysis

All data are presented as the mean ± SEM. Significant differences were determined by a two-way analysis of variance (ANOVA) followed by Tukey’s *post hoc* test. *P* < 0.05 was considered statistically significant.

## Results

### Central Infusion of Superoxide Scavenger TEM Prevents CIH-Induced Hypertension and Sympathetic Activation

**FIGURE 2:**
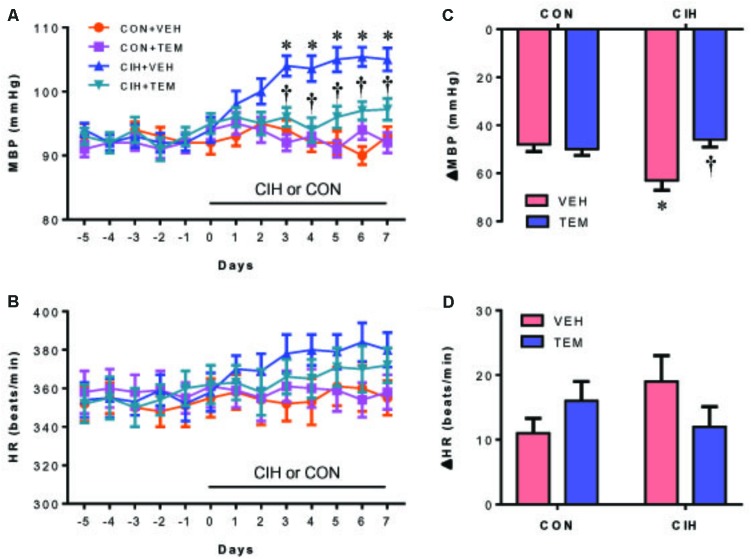
Mean blood pressure (MBP) **(A)** and heart rate (HR) **(B)**, and maximal changes (Δ) in MBP **(C)** and HR **(D)** following administration of ganglionic blocker hexamethonium, in control (CON) rats and chronic intermittent hypoxia (CIH)-exposed rats pretreated with intracerebroventricular (ICV) infusion of vehicle (VEH) or superoxide scavenger tempol (TEM). All data are presented as mean ± SEM (*n* = 8 for each group). ^∗^*P* < 0.05 vs. CON+VEH; ^†^*P* < 0.05, CIH+TEM vs. CIH+VEH.

Compared with CON+VEH rats, CIH+VEH rats exhibited a significantly increase in MBP after 3 days of CIH exposure and this increase persisted throughout the remaining 4 days of the CIH protocol (**Figure 2A**). Central infusion of superoxide scavenger TEM did not alter MBP in CON rats, but completely prevented the increase in MBP in CIH rats. There was no significant change in HR among four experimental groups throughout the experimental protocol (**Figure 2B**).

Blockade of ganglionic transmission with hexamethonium caused a significant reduction in MBP in all four groups (**Figure 2C**). Of note, the decrease in MBP was significantly greater in CIH+VEH rats than in CON+VEH rats. When compared with CIH+VEH rats, CIH+TEM rats had a significantly smaller depressor response. CON+TEM rats had a depressor response similar to CON+VEH rats. HR was not significantly altered by hexamethonium injection in all four experimental groups, although a slight increase was observed after hexamethonium injection (**Figure 2D**).

### Oxidative Stress and Inflammation Are Augmented in the PVN of CIH Rats but Are Inhibited by Central Infusion of Superoxide Scavenger TEM

**FIGURE 3:**
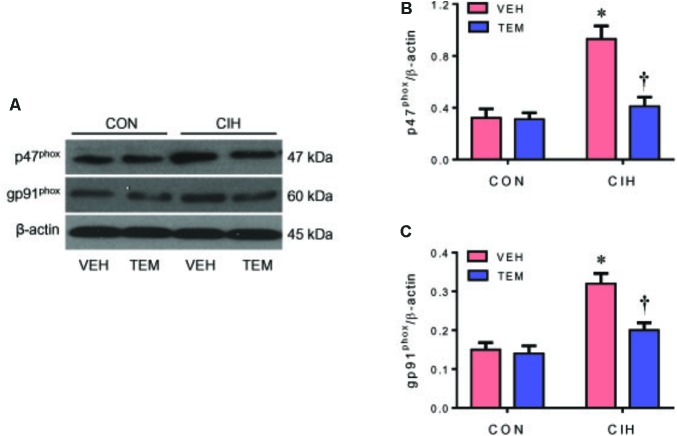
Representative Western blots **(A)** and statistical comparison of protein levels for p47^phox^ **(B)** and gp91^phox^ **(C)**, two important NAD(P)H oxidase subunits for reactive oxygen species (ROS) generation, in the hypothalamic PVN in CON rats and CIH-exposed rats pretreated with ICV infusion of VEH or superoxide scavenger TEM. All data are presented as mean ± SEM (*n* = 8 for each group). ^∗^*P* < 0.05 vs. CON+VEH; ^†^*P* < 0.05, CIH+TEM vs. CIH+VEH.

**FIGURE 4:**
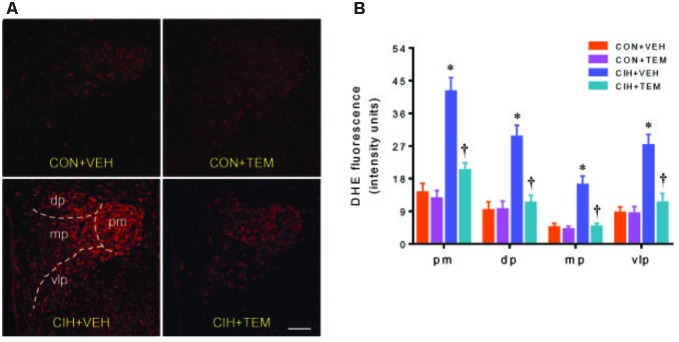
Representative dihydroethidium (DHE) images **(A)** and statistical comparison of DHE fluorescence **(B)** in four subregions of the hypothalamic PVN in CON rats and CIH-exposed rats pretreated with ICV infusion of VEH or superoxide scavenger TEM. All data are presented as mean ± SEM (*n* = 5 for each group). ^∗^*P* < 0.05 vs. CON+VEH at the same subregion; ^†^*P* < 0.05, CIH+TEM vs. CIH+VEH at the same subregion. pm, posterior magnocellular; dp, dorsal parvocellular; mp, medial parvocellular; vlp, ventrolateral parvocellular. Scale bar = 100 μm.

NAD(P)H oxidase–derived ROS in the central nervous system has been implicated in various forms of hypertension and other cardiovascular diseases. NAD(P)H oxidase can be activated through phosphorylation of protein kinase C (Rastogi et al., 2016). CIH has been shown to increase phosphorylation of protein kinase C and induce NAD(P)H oxidase activity in the central nervous system (Peng et al., 2009; Elliot-Portal et al., 2018). To examine whether oxidative stress plays a role in mediating blood pressure in CIH, we measured expression of NAD(P)H oxidase subunits p47^phox^ and gp91^phox^ in the PVN. Compared with CON+VEH rats, CIH+VEH rats had significant increases in protein levels of p47^phox^ (**Figures 3A,B**) and gp91^phox^ (**Figures 3A,C**) in the PVN, two important subunits of NAD(P)H oxidase for intracellular ROS generation. These increases were significantly inhibited by central infusion of TEM, which had no effects in CON rats. Moreover, intracellular ROS generation was detected using DHE staining. As shown in **Figure 4**, DHE fluorescence was augmented throughout the PVN in CIH+VEH rats, including posterior magnocellular, dorsal parvocellular, medial parvocellular, and ventrolateral parvocellular regions, compared with CON+VEH rats. Central infusion of TEM in CIH rats normalized DHE fluorescence in all subregions of the PVN. There was no difference between CON groups in DHE fluorescence in any subregion of the PVN.

**FIGURE 5:**
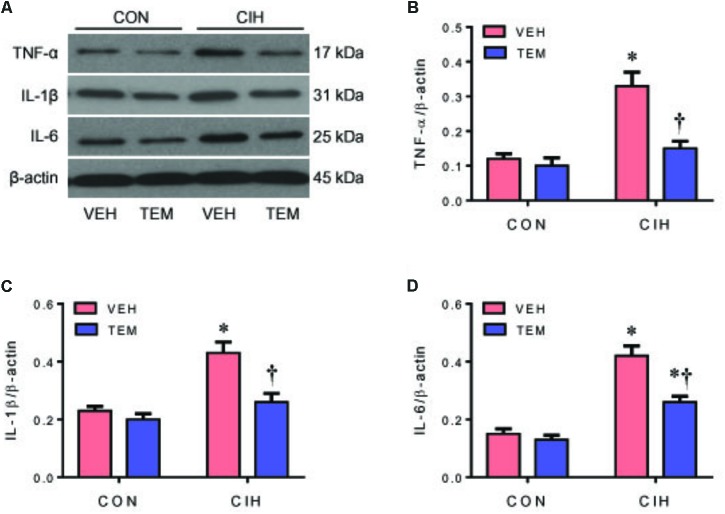
Representative Western blots **(A)** and statistical comparison of protein levels for pro-inflammatory cytokines tumor necrosis factor (TNF)-α **(B)**, interleukin (IL)-1β **(C)**, and IL-6 **(D)** in the hypothalamic PVN in CON rats and CIH-exposed rats pretreated with ICV infusion of VEH or superoxide scavenger TEM. All data are presented as mean ± SEM (*n* = 8 for each group). ^∗^*P* < 0.05 vs. CON+VEH; ^†^*P* < 0.05, CIH+TEM vs. CIH+VEH.

The protein levels of the proinflammatory cytokines TNF-α (**Figures 5A,B**), IL-1β (**Figures 5A,C**), and IL-6 (**Figures 5A,D**) were significantly higher in the PVN of CIH+VEH rats compared with CON+VEH rats. After central infusion of TEM, the protein levels of TNF-α and IL-1β were normalized and the protein levels of IL-6 were significantly reduced in the PVN of CIH rats. There were no differences in protein levels of above proinflammatory cytokines in the PVN between CON groups.

### Neuronal Activity Is Increased in the PVN of CIH Rats but Is Reduced by Central Infusion of Superoxide Scavenger TEM

**FIGURE 6:**
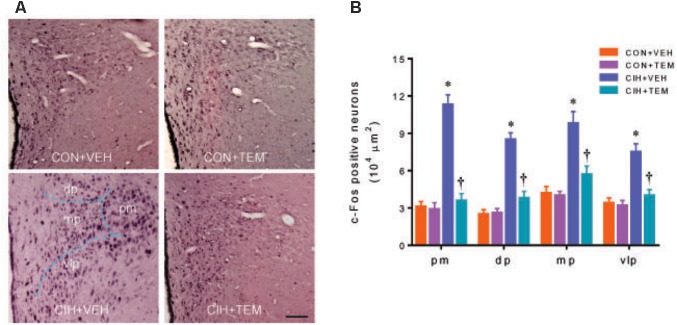
Representative images of c-Fos immunohistochemical staining **(A)**, a marker of neuronal excitation, and statistical comparison of c-Fos positive neurons **(B)** in four subregions of the hypothalamic PVN in CON rats and CIH-exposed rats pretreated with ICV infusion of VEH or superoxide scavenger TEM. All data are presented as mean ± SEM (*n* = 5 for each group). ^∗^*P* < 0.05 vs. CON+VEH at the same subregion; ^†^*P* < 0.05, CIH+TEM vs. CIH+VEH at the same subregion. pm, posterior magnocellular; dp, dorsal parvocellular; mp, medial parvocellular; vlp, ventrolateral parvocellular. Scale bar = 100 μm.

The expression of c-Fos activity has been used as a marker for acute neuronal activation in the central nervous system including the PVN (Hoffman et al., 1993; Abrams et al., 2010). Immunohistochemical study showed that there were fewer c-Fos positive neurons in the PVN of CON+VEH rats (**Figure 6**). The number of c-Fos positive neurons was increased in all four subregions of the PVN in CIH+VEH rats at 7 days after CIH, compared with CON+VEH rats. After central infusion of TEM, the number of c-Fos positive neurons in the PVN of CIH rats was reduced to the same extent as that observed in CON+VEH rats. The number of c-Fos positive neurons in the PVN was comparable between CON groups.

### Central Infusion of Superoxide Scavenger TEM Reduces Circulating Marker of Sympathetic Activation Without Alterations in Circulating Marker of Oxidative Stress and Inflammation

**FIGURE 7:**
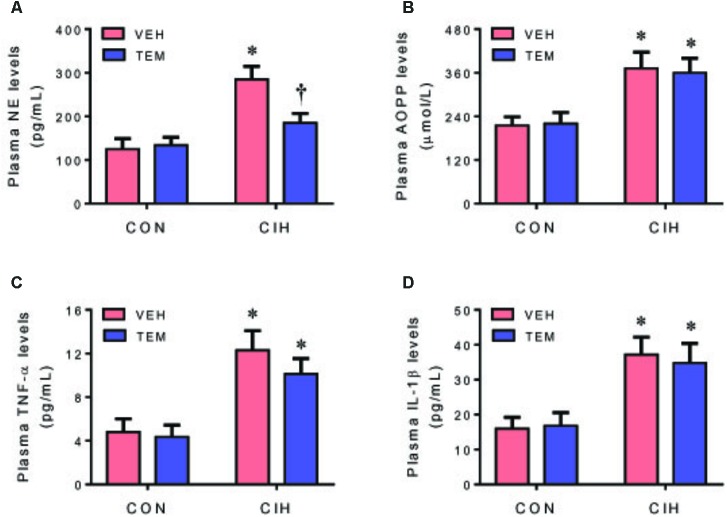
Plasma levels of norepinephrine (NE) **(A)**, a marker of sympathetic nerve activity, and plasma levels of advanced oxidation protein products (AOPP) **(B)**, a marker of systemic oxidative stress, and plasma levels of pro-inflammatory cytokines TNF-α **(C)** and IL-1β **(D)**, in CON rats and CIH-exposed rats pretreated with ICV infusion of VEH or superoxide scavenger TEM. All data are presented as mean ± SEM (*n* = 8 for each group). ^∗^*P* < 0.05 vs. CON+VEH; ^†^*P* < 0.05, CIH+TEM vs. CIH+VEH.

Plasma levels of NE (**Figure 7A**), a marker of sympathetic nerve activity, were higher in CIH+VEH rats as compared to CON+VEH rats. Central infusion of TEM significantly reduced plasma levels of NE in CIH rats but not in CON rats. Plasma levels of AOPP (**Figure 7B**), a marker of systemic oxidative stress, and plasma levels of TNF-α (**Figure 7C**) and IL-1β (**Figure 7D**) were also significantly elevated in CIH+VEH rats as compared to CON+VEH rats, but central infusion of TEM had no effects on circulating AOPP and proinflammatory cytokines in either CIH rats or CON rats.

## Discussion

The major findings of the present study are as follows: (1) rats exposed to CIH develop hypertension and exhibit augmented sympathetic nerve activity; (2) CIH exposure causes elevated oxidative stress and inflammation as well as increased neuronal activation in the PVN, a key brain region that regulates sympathetic outflow and blood pressure; (3) central infusion of superoxide scavenger TEM in rats exposed to CIH reduces oxidative stress, inflammation and neuronal activation in the PVN, leading to decreased sympathetic nerve activity and blood pressure; (4) central infusion of TEM doesn’t alter peripheral oxidative stress and inflammation. Taken together, these results suggest that CIH causes elevated oxidative stress and inflammation in the PVN, which are associated with CIH-induced sympathetic excitation and hypertension.

In the present study, rats exposed to CIH for 7 days developed a stable level of hypertension. Blockade of ganglionic transmission with hexamethonium caused a greater depressor response in CIH rats compared with CON rats. There was no significant difference in HR between CIH and CON rats. Plasma levels of NE were also markedly higher in CIH rats than CON rats. These results are consistent with previous reports (Sharpe et al., 2013; Faulk et al., 2017), indicating that augmented sympathetic nerve activity contributes to the development of hypertension under CIH conditions. Importantly, we found that CIH-induced hypertension was prevented by central infusion of superoxide scavenger TEM, suggesting that central oxidative stress mediates CIH-induced increase in blood pressure.

The carotid bodies are the principal peripheral chemoreceptors for detecting alterations in arterial blood oxygen concentration, and the resulting chemoreflex play an important role in regulating sympathetic tone, blood pressure and breathing (Prabhakar, 2016). Experimental studies in animals have shown that CIH induce chronic activation of carotid chemoreceptors, which may be due to sensitization of the carotid body response to hypoxia and sensory long-term facilitation (sLTF) in the glomus cell of the carotid body (Shell et al., 2016). Carotid body denervation can prevent CIH-induced sympathetic activation and hypertension (Lesske et al., 1997). In addition, activation of chemoreflex during CIH is also associated with altered signaling pathways in autonomic control centers in the brain (Shell et al., 2016). The PVN plays a critical role in controlling autonomic function under normal conditions and regulating cardiovascular activity in response to hypoxic stress (Shell et al., 2016). PVN neurons can be activated by hypoxia and play an important role in chemoreflex function (Berquin et al., 2000; King et al., 2013). Activation or disinhibition of the PVN induces increases in respiratory rate, blood pressure, and HR (Duan et al., 1997; Yeh et al., 1997; Schlenker et al., 2001). Stimulation of peripheral chemoreceptors leads to FosB/ΔFosB activity within PVN neurons, including presympathetic and magnocellular neurons (Smith et al., 1995; Berquin et al., 2000; Cruz et al., 2008). In contrast, lesion or blockade of the PVN attenuates ventilation and decreases arterial chemoreceptor-mediated responses including augmented sympathetic nerve activity and blood pressure (Olivan et al., 2001; Reddy et al., 2005). Experimental studies have shown that CIH exposure induces activation of neurons in the PVN, leading to increased blood pressure (Sharpe et al., 2013; Faulk et al., 2017). Chemical inhibition of neuronal activity in the PVN reduces sympathetic nerve activity and blood pressure in animals exposed to CIH (Sharpe et al., 2013). However, the precise mechanisms by which CIH induces activation of neurons in the PVN and increases sympathetic nerve activity are incompletely understood. New evidence reveals that excessive oxidative stress and inflammation in the cardiovascular regulatory centers of the brain, particularly in the PVN, lead to neuronal activation and augmented sympathetic nerve activity in various forms of hypertension and other cardiovascular diseases (Dange et al., 2015; Shen et al., 2015; Khor and Cai, 2017; Li Y. et al., 2017; Haspula and Clark, 2018; Jiang et al., 2018; Zhang et al., 2018). Microglia, the resident immune cells in the central nervous system, are often the major sources of pro-inflammatory cytokines in the central nervous system (Kiernan et al., 2016). Activation of microglia can increase expression of proinflammatory cytokines in the PVN, contributing to PVN neuronal activation (Zubcevic et al., 2017). Increase in NAD(P)H oxidase-dependent ROS have been demonstrated to induce microglia activity in the central nervous system under CIH conditions (Kiernan et al., 2016). Microglia-derived pro-inflammatory cytokines can in turn elevate oxidative stress (Snyder et al., 2017). These two components create a vicious cycle of oxidative stress and neuroinflammation facilitating each other and so leading to neuronal activation (Snyder et al., 2017). Interventions that reduce oxidative stress or inflammation in the PVN can attenuate neuronal activation and sympathetic nerve activity, preventing the development of high salt- or angiotensin II-induced hypertension (Shi et al., 2010; Bai et al., 2017; Haspula and Clark, 2018). CIH exposure in rats has recently been demonstrated to elevate oxidative stress and inflammation in the periphery and brain regions associated with neurodegeneration (Snyder et al., 2017). In the present study, we found that rats exposed to CIH for 7 days exhibited increased expression of NAD(P)H oxidases p47^phox^ and gp91^phox^ and augmented intracellular ROS generation (DHE staining), which were accompanied by increased expression of proinflammatory cytokines and neuronal excitation (c-Fos activity) diffusely throughout the PVN, involving neurons in both presympathetic and neuroendocrine regions of the PVN. Previous study has shown that central infusion of superoxide scavenger TEM reduces microglia activity and proinflammatory cytokine expression in the PVN and prevents angiotensin II-induced hypertension by inhibiting ROS production in the PVN (Jun et al., 2012). To determine the possible role of oxidative stress and inflammation in the PVN in mediating CIH-induced hypertension, we treated our animal model with ICV TEM. We observed that central TEM infusion in rats exposed to CIH reduced expression of NAD(P)H oxidases and intracellular ROS production in the PVN, along with decreased proinflammatory cytokines and neuronal excitability diffusely throughout the PVN. Moreover, rats exposed to CIH also had elevated systemic oxidative stress, inflammation and sympathetic nerve activity, as indicated by increased plasma levels of AOPP, proinflammatory cytokines and NE, respectively. Notably, central TEM infusion in rats exposed to CIH reduced sympathetic nerve activity but did not alter systemic oxidative stress and inflammation, suggesting the effects of central TEM infusion on oxidative stress and inflammation were limited in the brain. Collectively, these data indicate that CIH exposure induces elevated oxidative stress in both periphery and the brain, and that elevated oxidative stress in the brain causes neuroinflammation and neuronal excitation in the PVN of cardiovascular-regulatory center, which might lead to increased sympathetic nerve activity and hypertension under CIH conditions.

Nitric oxide, a well-known powerful cardiovascular-regulating agent, is involved in regulation of hypothalamic function. It has been demonstrated that CIH increases sympathetic nerve activity and blood pressure in part by downregulating neuronal nitric oxide synthase expression and reducing nitric oxide production in the PVN, where nitric oxide is sympathoinhibitory (Huang et al., 2007). CIH has been shown to reduce neuronal nitric oxide synthase expression and nitric oxide production in the PVN by decreasing NMDA receptor-mediated currents (Coleman et al., 2010). However, reduced nitric oxide production in the PVN is not observed until 35 days of CIH exposure (Coleman et al., 2010). It is unlikely that CIH-induced increases in sympathetic nerve activity and blood pressure in our study (only 7 days of CIH exposure) are due to reduction of nitric oxide in the PVN.

Viral tracing studies using pseudorabies virus or herpes simplex virus have demonstrated that the presympathetic neurons in the PVN are mainly presented in the dorsal parvocellular and ventrolateral parvocellular regions of the PVN (Affleck et al., 2012), suggesting that these two regions may be more relevant to regulation of sympathetic outflow. Previous studies have demonstrated that CIH exposure for 7 days in rats induces FosB/ΔFosB expression in the PVN, and the FosB/ΔFosB positive neurons are located mainly in the dorsal and medial parvocellular subnuclei (Knight et al., 2011; Knight et al., 2013). Using the same animal model, our study showed that CIH exposure caused c-Fos expression in the PVN, but the c-Fos positive neurons were located diffusely throughout the PVN. Because c-Fos activity that we measured in our study is a marker of acute neuronal activation and the interval between animal sacrifice and intermittent hypoxia is 1–3 h, it is possible that the increased PVN c-fos expression observed in our study might be due to the acute response of intermittent hypoxia. Additionally, parvocellular neurons in the PVN are key mediators of increased sympathetic nerve activity and blood pressure in CIH (Badoer, 2001; Shell et al., 2016). Vasopressin neurons in the PVN that regulate release of vasopressin are sympathoexcitatory, and activation of vasopressin receptors leads to inhibition of parasympathetic cardiac vagal neurons (Wang et al., 2002) and increased blood pressure (Kc et al., 2010). Whereas oxytocin neurons in the PVN release oxytocin that is cardioprotective and can reduce the adverse cardiovascular consequences of anxiety and stress (Jameson et al., 2016). Recent work has shown that activation of oxytocin neurons in the PVN prevents CIH-induced hypertension (Jameson et al., 2016). In the present study, we observed an overall neuronal activation (c-Fos expression) within the PVN after CIH exposure. Further studies are necessary to identify the activation of different phenotypes of neurons and determine which neurons might play a predominant role in mediating sympathoexcitation and hypertension under CIH conditions.

The present study has several limitations that should be noted. First, the present study focused on the roles of oxidative stress and inflammation in the PVN in mediating sympathetic nerve activity and blood pressure in rats exposed to CIH, and the superoxide scavenger TEM was administered by ICV infusion. We recognize that CIH-induced increases in sympathetic nerve activity and blood pressure cannot be attributed solely to elevated oxidative stress and inflammation in the PVN. ICV infusion of TEM would certainly have inhibited oxidative stress in other cardiovascular regulatory centers as well – the subfornical organ, where excessive oxidative stress has been shown to modulate blood pressure (Young et al., 2015). Indeed, Previous study has demonstrated that CIH exposure for 7 days induces neuronal activation as indicated by increased FosB/ΔFosB expression not only in the PVN, but also in the organum vasculosum laminae terminalis, median pre-optic nucleus and rostral ventrolateral medulla (Knight et al., 2011, 2013), suggesting that these brain regions are also involved in regulation of sympathetic nerve activity and blood pressure under CIH conditions. Second, sympathetic nerve activity was assessed by injection of hexamethonium bromide, which might block both sympathetic and parasympathetic activity in animals (Kurhanewicz et al., 2018). Further studies are necessary to provide the direct evidence that central tempol treatment attenuates sympathoexcitation in CIH by direct recording of sympathetic nerve activity. Third, angiotensin II infusion has been shown to induce oxidative stress, increase expression of angiotensin II type 1 receptor and proinflammatory cytokines in the PVN, contributing to PVN neuronal activation and hypertension (Kang et al., 2009). Central treatment with TEM attenuates oxidative stress, leading to decreases in expression of angiotensin II type 1 receptor and proinflammatory cytokines and neuronal activation in the PVN as well as blood pressure (Kang et al., 2009). CIH has recently been shown to increase plasma angiotensin II levels in animals (Lu et al., 2017) and central interventions to inhibit angiotensin II type 1 receptor in CIH animals reduce PVN neuronal activation and decrease hypertension (Shell et al., 2016). Since the renin-angiotensin system activity were not examined in this study, we could not exclude the possibility that CIH-induced elevation in angiotensin II accounts for increased angiotensin II type 1 receptor and proinflammatory cytokine expression as well as neuronal activation in the PVN, and that central TEM treatment of CIH rats reduces PVN neuronal activation and blood pressure in part by reducing central renin-angiotensin system activity in our study.

## Conclusion

In conclusion, the present study demonstrates that CIH induces elevated oxidative stress and inflammation in the PVN of the cardiovascular regulatory center which lead to PVN neuronal excitation and are associated with sympathetic excitation and increased blood pressure. The results from this study suggest that central oxidative stress and inflammation play an important role in the pathogenesis of CIH-induced hypertension. Targeting central oxidative stress and inflammation may be novel strategies for the prevention and treatment of hypertension in patients with OSA.

## Author Contributions

TL and BW conceived and designed the experiments. TL, YC, and CG performed the experiments and analyzed the data. TL wrote the paper.

## Conflict of Interest Statement

The authors declare that the research was conducted in the absence of any commercial or financial relationships that could be construed as a potential conflict of interest. The reviewer DZ and handling Editor declared their shared affiliation.
